# Age-dependent changes in fat- and water-soluble vitamins—National Health and Nutrition Examination Surveys study

**DOI:** 10.3389/fmed.2022.907067

**Published:** 2022-10-25

**Authors:** Ryszard Tomasiuk, Igor Z. Zubrzycki, Magdalena Wiacek

**Affiliations:** ^1^Faculty of Medical and Health Sciences, Kazimierz Pułaski University of Technology and Humanities in Radom, Radom, Poland; ^2^Department of Biometry and Mathematics, Botswana University of Agriculture and Natural Resources, Gaborone, Botswana

**Keywords:** aging, vitamins, health implications, reference range, health

## Abstract

Aging is an independent risk factor for the development of various diseases associated, among others, with detrimental blood levels of fat- and water-soluble vitamins. Thus, the objective of this study is to investigate age-related changes in blood levels of vitamin A, B12, C, D, and E. Subject serum vitamin levels were obtained from the combined National Health and Nutrition Examination Surveys (NHANES). NHANESIII and NHANES 1999–2000, 2001–2002, 2003–2004, and 2005–2006. The raw data set was stratified into five age groups G1- G5: 20 ≤ G1 < 30, 30 ≤ G2 < 40, 40 ≤ G3 < 50, 50 ≤ G4 < 60, and 60 ≤ G5 < 70 years of age. Age stratified data was cleaned using the modified Horn algorithm. The reference range for the vitamin level of a specific age group was defined as data between the first and third quartile of the subject defined by normal blood pressure and normal bone density. Age-dependent changes in serum/plasma vitamin levels were assessed using the bootstrap technique with 10,000 repeats and Bonferroni adjustment. There was a continuous increase in vitamin A, B12, D, and E levels in the blood. However, the vitamin C concentration remained virtually constant in all age groups. There was a lack of cross-correlations between lipid and water-soluble vitamin levels and blood pressure and bone health. The following reference levels for vitamin A, B12, C, D, and E in subjects older than 20 years of age were established: vitamin A: 1.32–2.8 mmol/L, vitamin B12: 257.94–498.33 pmol/L, vitamin C: 38.18–79.2 mmol/L, vitamin D: 76.33–199.36 nmol/L and vitamin E: 3.65–41.12 μmol/L.

## Introduction

The standard reference range for biochemical parameters of blood in humans should be derived from sampling the largest possible group of healthy people (1–3). However, analysis of the literature showed that representative samples involving more than a few hundred subjects have been relatively rare (4–7). It is rather surprising because deviations from the optimal concentration of biomolecules, such as, for example, vitamins, may indicate a disturbance of homeostasis (5, 8–13).

Deviation from normality in vitamin levels may result in the following medical complications: (a) age-related deficiency in vitamin D (14) manifests itself as an increased rate of bone fracture, (b) a deficiency in vitamin B12 (15) increases the risk of osteoporosis, cardiovascular diseases, a decrease in cognitive dysfunction, and dementia, (c) low level of vitamin C (16) leads to a decrease in collagen synthesis, resulting in increased fragility of blood vessels and arterial stiffness, (d) a lower level of vitamin E (17, 18) increases the risk of atherosclerosis, oxidative stress, cancer, cataract, and Alzheimer’s disease.

A recent study showed that aging is correlated with the reduced quality and quantity of meals consumed daily (19). This phenomenon induces a specific physiological response (20), resulting in an age-related decrease in fat metabolism that leads to an increased level of body fat tissue. Furthermore, it is often associated with dietary adjustment (21), reducing fat and sugar consumption, resulting in a decrease in the absorption of fat-soluble vitamins (22). Moreover, an age-related decrease in gastric acid production (23) also reduces vitamin bioavailability (24), and age-related dietary changes often decrease serum levels of ascorbic acid (vitamin C) and vitamin B12 (21, 25, 26).

Vitamin levels are also associated with the astronomical seasons (27–31). Seasonal changes in serum vitamin concentrations may influence hemodynamics, and blood pressure (32, 33), blood pressure-regulating hormone levels (34), biochemical indexes of bone turnover (35). However, generally accepted normal reference values for most physiological and biochemical parameters are independent of seasonal changes (36, 37). For example, the normal systolic blood pressure ratio (SBP) should be around 120 mmHg/80 mmHg (38), total serum cholesterol (TC), triglycerides (TG), high-density lipoprotein cholesterol (HDL-C), and low-density lipoprotein cholesterol levels (LDL-C), should adopt the following levels TC < 200 mg/dL, TG < 150 mg/dL, 40 mg/dL HDL-C < 60 mg/dL, LDL-C < 100 mg/dl (39).

## Materials and methods

### Study sample

Data used in this study were obtained from the National Health and Nutrition Examination Surveys (NHANES) conducted by the National Center for Health Statistics (NCHS) carried out in separate stages since 1971 (40). The purpose of NHANES is to gather information and monitor descriptively and quantitatively the physical state, disease, and interrelations of physiological and psychical conditions and nutrition in the population of the U.S.A (41).

NHANES III was conducted in two phases; phase 1: from 1988 to 1991 and phase 2: from 1991 to 1994. In 1999, the NCHS began to operate NHANES as a continuous survey collecting data (42). The measurements and laboratory tests reported in the NHANES surveys are carried out on representative randomly chosen subsamples (43). Subject selection for the data collection process was performed in a manner allowing to nullify the chance of repeated measurements across different NHNAES periods.

### Collecting biochemical and anthropometric parameters

Serum lipid levels (SLL), including TC, TG, and HDL-C, were measured using NHANES protocols. TC levels were measured enzymatically in serum or plasma in a series of coupled reactions that hydrolyze cholesterol esters and oxidize the 3-OH cholesterol group. TG levels were assessed using a series of coupled reactions in which triglycerides were hydrolyzed to produce glycerol. HDL-C was measured by precipitation of other lipoproteins with a polyanion/divalent cation mixture. The laboratory procedures are described on the NHANES web page: NHANESI III^1^ and NHANES 1999–2006.^2^ The LDL level was calculated using the Friedewald equation: LDL = TC–HDL–TG/5.

According to the NHANES laboratory manual Vitamin D, B12, C, A, and E levels were determined using the following procedures: (a) serum 25-hydroxyvitamin D [25(OH)D] (vitamin D) level was determined using Diasorin-Incstar 25(OH)D assay [which is the limitation of this report due to the variability of the results rendered by this method (44)], (b) vitamin B_12_ and serum folate (vitamin C) levels were measured using the Bio-Rad Laboratories “Quantaphase Folate” radioassay kit (45), (c) vitamin A (retinol) and vitamin E (α-tocopherol) were measured using isocratic high-performance liquid chromatography (HPCL) with detection at 300, 325, and 450 nm.

According to the NHANES manuals, bone mineral density (BMD) measurements were performed in the Mobile Examination Center using a Hologic QDR-1000 X-ray densitometer.

SBP and diastolic blood pressure (DBP) are the averages of three measurements provided by the NHANES data set.

Standing height was measured using a stadiometer with a fixed vertical backboard and an adjustable headpiece. Body mass was determined using digital weight. Subject body mass index (BMI) was calculated using the formula: BMI (kg/m^2^) = body mass (kg)/[standing height]^2^ (m^2^).

Further details on the adopted methodology can be found in the NHANES examination and laboratory protocols.^3^

The “raw” data sample was a join NHANESIII, NHANES 1999–2000, 2001—2002, 2003–2004, and 2005—2006 data set comprising *N* = 34,057 men and 36,113 women. The resulting data were stratified into five age groups (G1–G5): 20 ≤ G1 < 30, 30 ≤ G2 < 40, 40 ≤ G3 < 50, 50 ≤ G4 < 60, and 60 ≤ G5 < 70 and subjected to a data cleaning procedure.

### Data cleaning

**FIGURE 1:**
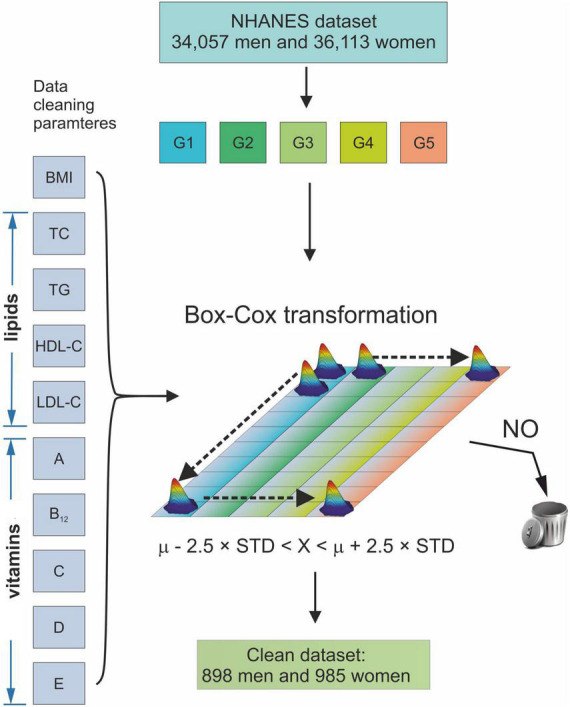
Schematic diagram of the data cleaning procedure. G1–G5 age groups: 20 ≤ G1 < 30, 30 ≤ G2 < 40, 40 ≤ G3 < 50, 50 ≤ G4 < 60, and 60 ≤ G5 < 70 years of age. BMI, Body Mass Index; TC, Total Cholesterol; TG, Triglycerides; HDL-C, High-Density Lipoprotein Cholesterol; LDL, Low-Density Lipoprotein Cholesterol; A, vitamin A; B12, vitamin B12; C, vitamin C; D, vitamin D; E, vitamin E; μ, mean of a sample; STD, standard deviation of a sample.

Figure 1 shows a flow chart of the data cleaning procedure. It was a two-step procedure. The first step was data selection, and the second step was data cleaning. In Step 1, all subjects with (a) missing age on the examination, (b) smoking tobacco with a frequency greater than or equal to one cigarette per day (46), (c) consuming alcohol with an amount greater than or equal to one alcoholic beverage per day (47), (d) undergoing chemotherapy treatment during the examination (48), (e) pregnant during the examination (49), (f) breastfeeding during the examination (50) or (g) younger than 20 years of age, were rejected from the study. In Step 2, all data that fall into the outliers of BMI, waist circumference (WC), SLL, and vitamin levels were deleted. This procedure used the modified Horn algorithm (51). Thus, a sample was subjected to a Box-Cox transformation to normality, and the outliers comprising values less than the mean –2.5 • STD or greater than the mean + 2.5 • STD were removed from the sample (52).

The clean data set comprised of 9994 non-Hispanic whites, 8,400 non-Hispanic black, 8,391 Mexican American, and 1,250 subjects of other races.

To accommodate technical differences in vitamin D levels assessment between NHANES III and NHANES 1999—2006, the following formula was used: NHANES III, reformulated RIA test of 25 (OH) D = [0.8429* NHANES III 25(OH)D original RIA assay] + 2.5762 nmol/L (*r* = 0.8966) (53).

### Definition of osteoporosis and hypertension

Following the requirements of the World Health Organization (WHO), osteopenia and osteoporosis were defined as BMD adopting values 1 < μ ≤ 2.5 and μ > 2.5 STD from average healthy subjects (54).

Hypertension was defined according to the Guideline for the Prevention, Detection, Evaluation, and Management of High Blood Pressure in Adults (55). Therefore, the following rule was used for the classification of blood pressure (BP) (55)/Thus, normotensive subjects are defined by SBP < 120 and DBP < 80 mmHg, subjects with elevated blood pressure 120 ≤ SBP ≤ 129 and DBP < 80, subjects with hypertension Stage 1: 130 ≤ SBP ≤ 139, or 80 ≤ DBP ≤ 89; subjects with hypertension Stage 2: SBP ≥ 140, or DBP ≥ 90 mmHg.

### Statistical analysis

All statistical calculations were calculated using the R programming environment (56). The normality of sample distribution was verified using the Shapiro-Wilk (57) test. Hypothesis testing was performed at the significance level α of 0.05. The reference range for vitamins was defined as an interval between the first and third quartiles of the respective data. Differences in means for specific age groups were tested using a bootstrapped test for differences in means of 10,000 repetitions with replacement. In the longitudinal analysis, an analogous approach was used. However, to accommodate for multiplicity, Bonferroni adjustment was employed.

## Results

Reference values for serum/plasma vitamin levels for the multiethnic sample comprising *N* = 898 male and 985 female subjects are gathered in Table 1.

**TABLE 1:** Reference interval of blood concentration of vitamin A (μmol/L), B12 (pmol/L), C (μmol/L), D (nmol/L), and E (M mol/L) concentrations stratified by age group in men.

	*n*	Age group	Vitamin A		Vitamin B_12_		Vitamin C		Vitamin D		Vitamin E	
			1st Qrt	3rd Qrt	1st Qrt	3rd Qrt	1st Qrt	3rd Qrt	1st Qrt	3rd Qrt	1st Qrt	3rd Qrt
MEN	2,663	G1	1.66	2.07	283.39	413.28	39.70	66.40	87.21	149.51	4.66	20.34
	2,509	G2	1.86	2.44	273.06	445.01	42.00	63.85	112.15	183.78	4.55	25.96
	2,294	G3	1.83	2.5	296.12	426.75	43.43	61.45	105.91	161.99	5.25	23.81
	1,710	G4	1.99	2.54	305.17	464.94	48.00	67.60	118.36	183.78	4.58	28.72
	5,439	G5	1.91	2.75	262.73	459.96	38.18	67.45	109.03	172.87	5.37	29.43
WOMEN	2,832	G1	1.32	2.01	264.02	449.45	42.15	73.20	76.33	160.42	4.35	19.89
	2,837	G2	1.39	2.12	273.62	498.33	41.85	71.00	87.21	174.45	4.14	26.04
	2,564	G3	1.41	1.88	257.94	438.00	46.28	69.00	93.45	161.99	4.28	27.13
	1,950	G4	1.77	2.28	267.16	438.01	54.2	79.20	121.48	174.45	4.3	30.91
	5,900	G5	1.99	2.80	282.66	494.09	46.85	76.95	121.48	199.36	3.65	41.12
Reference range			1.32	2.80	257.94	498.33	38.18	79.20	76.33	199.36	3.65	41.12

G1–G5 correspond to age groups: 20 ≤ G1 < 30, 30 ≤ G2 < 40, 40 ≤ G3 < 50, 50 ≤ G4 < 60, and 60 ≤ G5 < 70.

**FIGURE 2:**
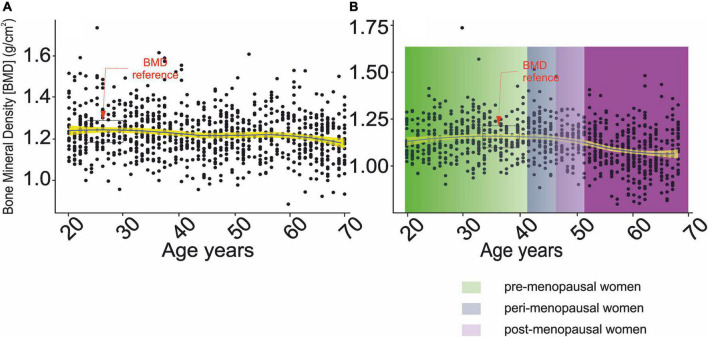
Changes in bone mineral density as a function of chronological age. **(A)** Men, **(B)** women. 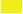 – denotes confidence interval for the mean, 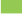, 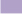, and 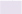 correspond to pre-, peri- and post-menopausal period (58).

The BMD values for defining normal bone health were obtained by analyzing age-related changes in BMD (Figures 2A,B). Therefore, the mean and standard deviation (STD) of BMD were used in the age range between 23 and 30 years of age and 33–40 years of age in men and women, respectively. The mean and STD for men and women were equal to 1.22 ± 0.11 g/cm^2^ and 1.23 ± 0.11 g/cm^2^, respectively.

**FIGURE 3:**
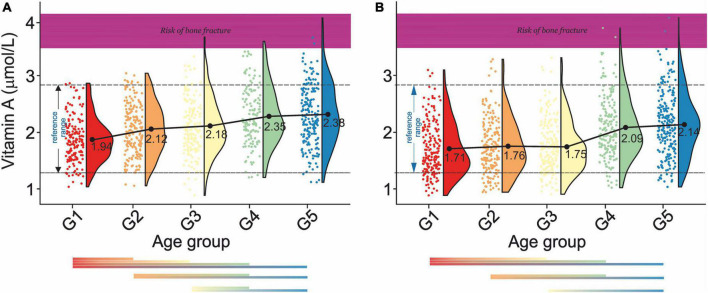
Rain-cloud plots of age-dependent changes in vitamin A levels as a function of age group G1: 20 ≤ 1 < 30, G2: 30 ≤ 2 < 40, G3: 40 ≤ 3 < 50, G4: 50 ≤ 4 < 60, and 60 ≤ G5 < 70 years of age-stratified by gender **(A)** men, **(B)** women. Mean values for a specific sample are truncated to one decimal place. Color bars refer to statistically significant differences between groups at *P* < 0.05. Shaded area corresponds to vitamin A levels at which an increased risk of bone fracture is observed (98).

**FIGURE 4:**
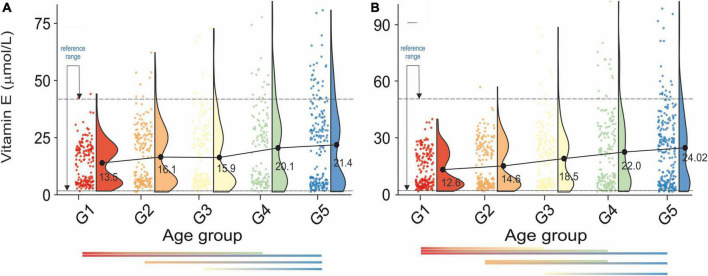
Rain-cloud plots of age-dependent changes in vitamin E levels as a function of age group G1: 20 ≤ 1 < 30, G2: 30 ≤ 2 < 40, G3: 40 ≤ 3 < 50, G4: 50 ≤ 4 < 60, and 60 ≤ G5 < 70 years of age-stratified by gender **(A)** men, **(B)** women. Mean values for a specific sample are truncated to one decimal place. Color bars refer to statistically significant differences between groups at *P* < 0.05.

Changes in mean serum levels of vitamins A, B_12_, C, D, and E as a function of age group, osteoporosis, and blood pressure for men and women are compiled in Tables 2A,B, respectively. Figures 3A,B, 4A,B illustrate the longitudinal changes in vitamin levels for men and women, respectively.

**TABLE 2A:** Mean levels of vitamin A (μmol/L), B12 (pmol/L), C (μmol/L), D (nmol/L), and E (μmol/L) concentrations stratified by age group and osteoporosis status in women.

	Age group	Vitamin A	Vitamin B12	Vitamin C	Vitamin D	Vitamin E	Bone health
MEN	G1	1.95	365.39	54.14	48.3	13.27	Normal
		1.86	343.46	55.59	44.2	15.95	Osteopenia
	G2	2.12	383.55	52.66	53.7	16.05	Normal
		2.17	361.75	48.57	53.8	16.82	Osteopenia
	G3	2.19	381.05	46.6	48.1	15.26	Normal
		2.11	424.68	53.35	41.9	19.74	Osteopenia
	G4	2.34	395.73	53.56	55.2	19.22	Normal
		2.41	342.82	52.06	50.6	24.99	Osteopenia
		1.53	455.35	37.5	49.1	27.75	Osteoporosis
	G5	2.42	408.50	56.96	52.5	20.96	Normal
		2.24	383.18	54.25	50.4	23.85	Osteopenia
		2.36	226.81	62.27	39.5	15.10	Osteoporosis
WOMEN	G1	1.73	374.3	57.92	48.3	12.54	Normal
		1.62	365.83	49.35	44.2	10.72	Osteopenia
	G2	1.77	399.88	56.00	53.7	14.33	Normal
		1.77	430.82	48.26	53.8	15.81	Osteopenia
	G3	1.74	390.16	54.18	48.1	18.21	Normal
		1.83	350.18	53.81	41.9	22.92	Osteopenia
	G4	2.09	418.9	61.73	55.2	21.28	Normal
		2.09	437.11	57.18	50.6	25.48	Osteopenia
		1.65	356.95	30.07	49.1	36.91	Osteoporosis
	G5	2.13	425.39	57.96	52.5	24.31	Normal
		2.16	419.30	61.00	50.4	24.17	Osteopenia
		1.93	447.47	36.72	39.5	23.71	Osteoporosis

**TABLE 2B:** Mean serum concentration of vitamin A (μmol/L), B12 (pmol/L), C (μmol/L), D (nmol/L), and E (μmol/L) concentrations stratified by age group and blood pressure in women.

	Age group	Vitamin A	Vitamin B12	Vitamin C	Vitamin D	Vitamin E	Blood pressure
MEN	G1	1.89	363.9	54.77	50.71	13.61	Normal
		2	371.53	56.35	49.86	12.34	Elevated
		2.04	201.11	35.8	37.44	12.38	Hypertension stage 1
		2.17	247.48	44.87	72.38	5.41	Hypertension stage 2
	G2	2.15	374.61	52.35	56.84	16.57	Normal
		2.06	369.84	54.81	52	14.19	Elevated
		2.25	420.45	49.8	53.49	14.78	Hypertension stage 1
		1.87	268.44	42.58	48.67	18.37	Hypertension stage 2
	G3	2.22	408.43	50.19	56.89	15.63	Normal
		2.22	444.48	46.1	57.24	15.08	Elevated
		2.04	334.31	45.56	60.32	20.96	Hypertension stage 1
		1.97	436.8	38.76	42.43	14.32	Hypertension stage 2
	G4	2.34	397.65	56.98	62.66	19.94	Normal
		2.42	412.05	58.2	55.85	20.11	Elevated
		2.28	437.22	45.73	49.92	17.46	Hypertension stage 1
		2.03	358.83	36.09	49.92	15.76	Hypertension stage 2
	G5	2.33	357.54	57.82	57.41	20.63	Normal
		2.42	479.24	64.14	61.01	25.53	Elevated
		2.56	361.53	64.38	58.66	28.89	Hypertension stage 1
		2.35	581.34	52.92	59.22	21.87	Hypertension stage 2
WOMEN	G1	1.67	374.36	57.42	49.23	12.27	Normal
		1.78	350.23	58.04	42.79	11.2	Elevated
	G2	1.77	402.6	55.4	55.04	14.78	Normal
		1.71	375.84	61.47	45.93	18.14	Elevated
		2.56	195.57	49.4	89.86	27.56	Hypertension stage 1
	G3	1.71	389.56	56.8	51.55	17.95	Normal
		1.65	398.68	53.73	43.41	16.04	Elevated
		1.65	480.86	52.31	43.86	7.66	Hypertension stage 1
		2.14	354.17	42.22	36.53	20.41	Hypertension stage 2
	G4	2.09	389.83	66.23	58.27	17.71	Normal
		1.93	382.57	61.63	52.66	23.86	Elevated
		2.21	462.32	51.47	50.47	20.92	Hypertension stage 1
		2.73	485.97	57.56	51.17	33.44	Hypertension stage 2
	G5	2.27	410.69	63.61	60.78	23.28	Normal
		2.28	482.13	67.6	51.79	16.25	Elevated
		1.98	666.5	64.3	49.3	12.67	Hypertension stage 1
		2.16	313.97	45.46	55.98	28.81	Hypertension stage 2

G1–G5 correspond to age groups: 20 ≤ G1 < 30, 30 ≤ G2 < 40, 40 ≤ G3 < 50, 50 ≤ G4 < 60, and 60 ≤ G5 < 70 years of age.

Analysis of changes of serum vitamin A level in men (Figure 3A) showed a gradual increase (G1:1.94–G5:2.38 μmol/L) with statistically significant differences between G1- G2, G1–G3, G1- G4, and G1–G5, G2–G4, and G2–G5, and G3–G4, G3–G5. In women (Figure 3B), a continuous decrease in serum vitamin A levels between G1 and G5 (1.71–2.14 mol/L) was observed. Statistically, significant differences were also observed between G1-G2, G1-G3, G1-G4, G1-G5, G2-G4, G2-G5, and G3-G5. There was a lack of apparent correlations between serum vitamin A level and normal, osteopenic, and osteoporotic status in all age groups in both sexes (Table 2A). However, in G3 and G4, a decrease in vitamin A concentration was observed between osteopenic and osteoporotic subjects in men and women. There was no correlation between vitamin A levels and blood pressure (Table 2B). However, in women in the G1–G4 groups, hypertension stage 1 was defined by higher levels of vitamin A than hypertension stage 2.

**FIGURE 5:**
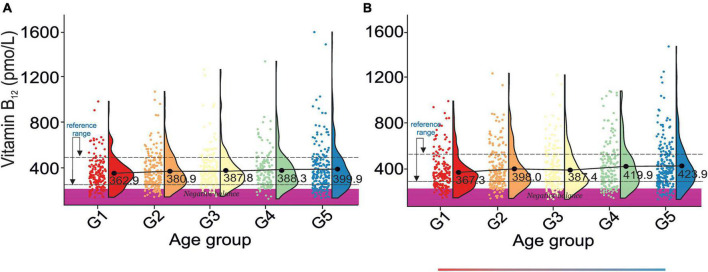
Rain-cloud plots of age-dependent changes in vitamin B12 levels as a function of age group G1: 20 ≤ 1 < 30, G2: 30 ≤ 2 < 40, G3: 40 ≤ 3 < 50, G4: 50 ≤ 4 < 60, and 60 ≤ G5 < 70 years of age-stratified by gender **(A)** men, **(B)** women. Mean values for a specific sample are truncated to one decimal place. Color bars refer to statistically significant differences between groups at *P* < 0.05. Shaded area corresponds to vitamin B12 negative balance levels (82).

There was a gradual increase in serum vitamin B_12_ levels between G1 and G5 in both sexes, Figures 5A,B. Vitamin B_12_ levels are not correlated with bone health (Table 2A). However, osteoporotic subjects are defined by a lower vitamin B12 level than osteopenic subjects. Additionally, vitamin B_12_ levels do not correlate with blood pressure (Table 2B). However, the difference between stages 1 and 2 of hypertension results in a pronounced change in serum vitamin B_12_ levels.

**FIGURE 6:**
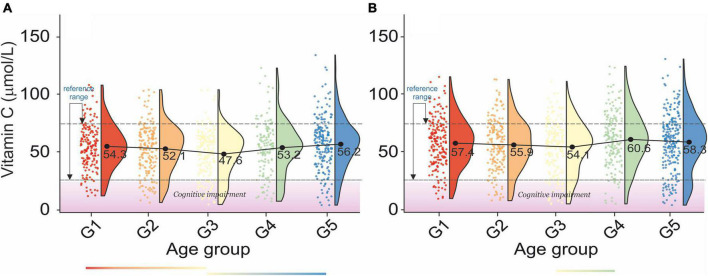
Rain-cloud plots of age-dependent changes in vitamin C levels as a function of age group G1: 20 ≤ 1 < 30, G2: 30 ≤ 2 < 40, G3: 40 ≤ 3 < 50, G4: 50 ≤ 4 < 60, and 60 ≤ G5 < 70 years of age-stratified by gender **(A)** men, **(B)** women. Mean values for a specific sample are truncated to one decimal place. Color bars refer to statistically significant differences between groups at *P* < 0.05. A shaded area corresponds to an increased risk of cognitive impairment vitamin levels (86).

Analysis of age-dependent changes in serum vitamin C levels in men and women revealed two distinct periods. First, a decrease between G1–G3 and second an increase between G3–G5, Figures 6A,B. Although there were no clear correlations between bone health and serum vitamin C levels (Table 2A), osteopenic and osteoporotic subjects are defined by markedly different serum vitamin C concentrations. Furthermore, an examination of Table 2B revealed that serum vitamin C levels decreased with an increase in blood pressure in both sexes.

**FIGURE 7:**
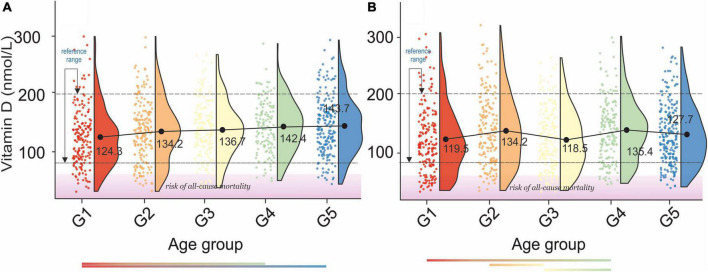
Rain-cloud plots of age-dependent changes in vitamin D levels as a function of age group G1: 20 ≤ 1 < 30, G2: 30 ≤ 2 < 40, G3: 40 ≤ 3 < 50, G4: 50 ≤ 4 < 60, and 60 ≤ G5 < 70 years of age-stratified by gender **(A)** men, **(B)** women. Mean values for a specific sample are truncated to one decimal place. Color bars refer to statistically significant differences between groups at *P* < 0.05. Shaded area corresponds to increased risk of all-cause mortality (91).

A gradual increase in serum vitamin D levels was observed as a function of age in both genders, Figures 7A,B. A decrease between G2–G3 and an increase between G3–G4, corresponding to menopausal transition (58), was observed in women. In men, changes in serum vitamin D did not directly reflect bone health status, Table 2A. However, the difference between osteopenic and osteoporotic subjects was always associated with a distinct decrease in serum vitamin D levels. A decrease in serum vitamin D was observed among normal, osteoporotic, and osteoporotic women. No cross-correlations were observed between vitamin D levels and blood pressure in both sexes (Table 2B).

A continuous increase in serum vitamin E levels was observed as a function of the age group, Figures 4A,B. Moreover, analysis of Figures 4A,B unfolded bimodality in age groups G1-G3. At present we are unable to explain the observed phenomenon. However, similar behavior was already reported in the study (59). Although there was no cross-correlation between serum vitamin E and bone health in either sex (Table 2A), the difference between osteopenic and osteoporotic status was reflected by a distinct change in serum vitamin E levels. Furthermore, changes in serum vitamin E level did not correspond directly to blood pressure, Table 2B. However, in women, a transition between stages 1 and 2 of hypertension was associated with a marked increase in serum vitamin E levels.

## Discussion

Under normal physiological conditions, minimum amounts of vitamins are critical to homeostasis (60). Some studies have shown that small changes in serum vitamin levels, caused, among others, by seasonal variations, can negatively affect human health (61). Despite that, only the Australian government has advised to include seasonal vitamin variation in clinical practice (62). Due to the mobility of the world’s population and population subgroups *per se* (63), following seasonal changes in serum vitamin levels has limited medical value.

The strength of the presented study is a sample size of studied age groups resulting in significant strength of the elucidated statistical references. The limitation of this study is the technology of assessment of the levels of serum 25-hydroxyvitamin D (25(OH)D) (vitamin D) using Diasorin-Incstar 25(OH)D assay.

Studies on correlations between blood vitamin levels and human health showed that vitamin C deficiency (< 11.4 mol/L) is associated with a higher risk of coronary heart disease (16). Vitamin E supplementation in the form of α-tocopherol plays a positive role in inflammation (64) and protects the skin against sun radiation (65). Vitamin B_12_ intake inhibits cognitive decline, macular degeneration and maintains bone health (66). Vitamin A deficiency increase the risk of occurrences of measles and diarrhea (67), whileacute and chronic effects of vitamin A toxicity are well documented (68). Vitamin D is essential for preventing osteoporosis, prostate, colon, and breast cancer (69). It is also inversely related to hypertension (70) and type I diabetes mellitus (71).

Although a variety of reports discussed the medical manifestations of changes in serum/plasma levels of vitamin C (72), vitamin D (73), vitamin B_12_ (74), vitamin C (72), and vitamins A and E (7) in human health, there is a lack of information changes in their levels as a function of age and the respective reference range. Additionally, the results presented depend on the data source.

For example, Merck (75) provided a gender-independent standard range of vitamin A between 0.98 and 3.00 μmol/L, while de Pee and Dary (76) reported a range between 1.13 and 2.63 μmol/L and 1.32–2.44 μmol/L for men and women, respectively. Although these differences are subtle, they may have profound health implications. For example, Wu et al. (77) reported an increased risk of bone fracture in males with serum/plasma vitamin A concentrations greater than 1.40 mol/L.

Our report unfolded a distinct age-dependent increase in serum vitamin A levels in both men and women. With the respective gender-independent reference between 1.32 and 2.80 μmol/L. The results obtained correspond well to the values previously reported (78). Cross-correlation of our data with those on a bone fracture as a function of serum retinol levels (77) revealed that aging is associated with an increase in the number of subjects at risk of bone fracture (Figures 3A,B). This study confirmed a positive association between serum vitamin A levels and blood pressure levels (79) in G1 and G1–G5 in male and female subjects. However, men over 30 years of age were defined by the negative association between serum vitamin A levels and hypertension.

The reference range for serum vitamin B_12_ levels derived from this study was 257.94–498.33 pmol/L and roughly corresponded to the previously reported values (5, 74, 80, 81). However, apparent differences in vitamin B_12_ levels between sources are, in our opinion, caused by methodological and ethnic variations among studies. The cross-correlation of our results with those reported by Herber (82) revealed 20% of men and women in all age groups defined by a negative vitamin B_12_ balance (< 221 pmol/L). Such a large percentage of randomly selected subjects falling into the negative vitamin B_12_ balance may indicate: (1) a grave vitamin B_12_ supplementation problem in the U.S.A, (2) the need for amendment of negative vitamin B_12_ balance cut-off values.

The age and sex-independent plasma vitamin C reference range was 38.18–79.2 μmol/L. Our results are more restricted than those of Hagel et al. (28.41–85.23 μmol/L) (83). Furthermore, the lower bracket of the vitamin C reference range is less than that reported by Zempleni (45–80 μmol/L) (84). However, Tietz’s textbook on molecular diagnostics (85) provided a serum vitamin C range between 23 and 85μmol/L, while the Royal College of Pathologists of Australasia provides a range between 30 and 80μmol/L. The cross-correlation of the results of this study with those on ascorbic acid levels in blood plasma and neuropsychiatric effects (86) revealed that ∼ 25% of the study sample in each age group had an increased risk of cognitive impairment (vitamin C levels < 27.82μmol/L). Our report also revealed the lack of cross-correlation between blood vitamin C levels and bone health in older women. However, there is a marked decrease in vitamin C levels between osteopenic and osteoporotic subjects. In this regard, our report confirmed the previous study (87). Furthermore, the results presented validated the previously reported inverse association between plasma vitamin C and BP (88).

The serum vitamin D reference interval established by this study was 76.33–199.36 nmol/L. The values obtained correlate well with previously reported “optimal” gender, and age-independent reference ranges for serum vitamin D levels in the populations other that the USA (89) 74.88–249.6 nmol/L and 62.4–199.68 nmol/L for USA population (90). An amalgam of the study on the age of menopausal transition with the results of this study indicated that the pre-, peri-, and post-menopausal transition is associated with a decrease in serum vitamin D levels. Furthermore, the cross-correlation of the results of this report with the study on 25-hydroxyvitamin D levels and mortality risk revealed that 10% of the studied population was at risk of all-cause mortality (< 44.43 nmol/L) (91). Although male osteopenic subjects were defined by slightly higher vitamin D levels than normal subjects, osteoporosis is always manifested by a distinct drop in vitamin D levels compared to normal and osteopenic subjects. The later observation is confirmed by the latest study of LeBoff et al. (92). Moreover, the latter study unfolded that supplementation with vitamin D3 (2,000 IU per day) without co-administering calcium did not result in a lower risk of fractures.

Analysis of blood pressure as a function of serum vitamin D concentration unfolded, as in another study (93), lack of distinctive correlations in elderly subjects; see age groups G4 and G5 in men and women, Figures 7A,B. However, the difference between stage 1 hypertension and stage 2 hypertension is associated with a distinct decrease in vitamin D levels. This observation was confirmed by the study of Zhou et al. (94).

The standard reference range of serum vitamin E levels unfolded in this study was 3.65–41.12μmol/L. This result is much stricter than previously reported by Ford et al. (0.8–71.9 μmol/L) (95). Therefore, although the previous study indicated a potential positive role for serum vitamin E in maintaining BMD (96), this study does not support this conclusion. Furthermore, we were unable to confirm the positive and significant association between serum vitamin E with SBP and DBP. Nevertheless, subjects with stage 2 hypertension are defined by a lower vitamin E concentration than the subjects with stage 1 hypertension.

## Conclusion

The results of this study unfolded the following vitamin ranges for subjects older than 20 years: vitamin A: 1.32–2.8 mmol/L, vitamin B_12_: 257.94–498.33 pmol/L, vitamin C: 38.18–79.2 mmol/L, vitamin D: 76.33–199.36 nmol/L, and vitamin E: 3.65–41.12 μmol/L. Furthermore, our results did not confirm direct correlations between cardiovascular—and bone- health and blood vitamin levels. The observed small trend in an increase in serum levels of studied vitamins as a function of age combined with the results of the study on vitamins intakes among elderly (97) confirm that a majority of elderly support their diet with vitamin supplements.

## Data availability statement

Publicly available datasets were analyzed in this study. This data can be found here: https://wwwn.cdc.gov/nchs/nhanes/Default.aspx.

## Author contributions

All authors listed have made a substantial, direct, and intellectual contribution to the work, and approved it for publication.

## Footnotes

1
https://www.cdc.gov/nchs/nhanes/nh3rrm.htm

2
https://wwwn.cdc.gov/nchs/nhanes/continuousnhanes/manuals.aspx?BeginYear=1999

3
https://www.cdc.gov/nchs/nhanes/about_nhanes.htm
